# Supplementary figures and images for: Body Length and Craniometrics of Non-Native Raccoons in Two Regions in Middle Japan during Early Invasion Stages
Source: Animals (Basel). 2022 Dec 23;13(1):55. doi: 10.3390/ani13010055 (PMC9817695; doi:10.3390/ani13010055)

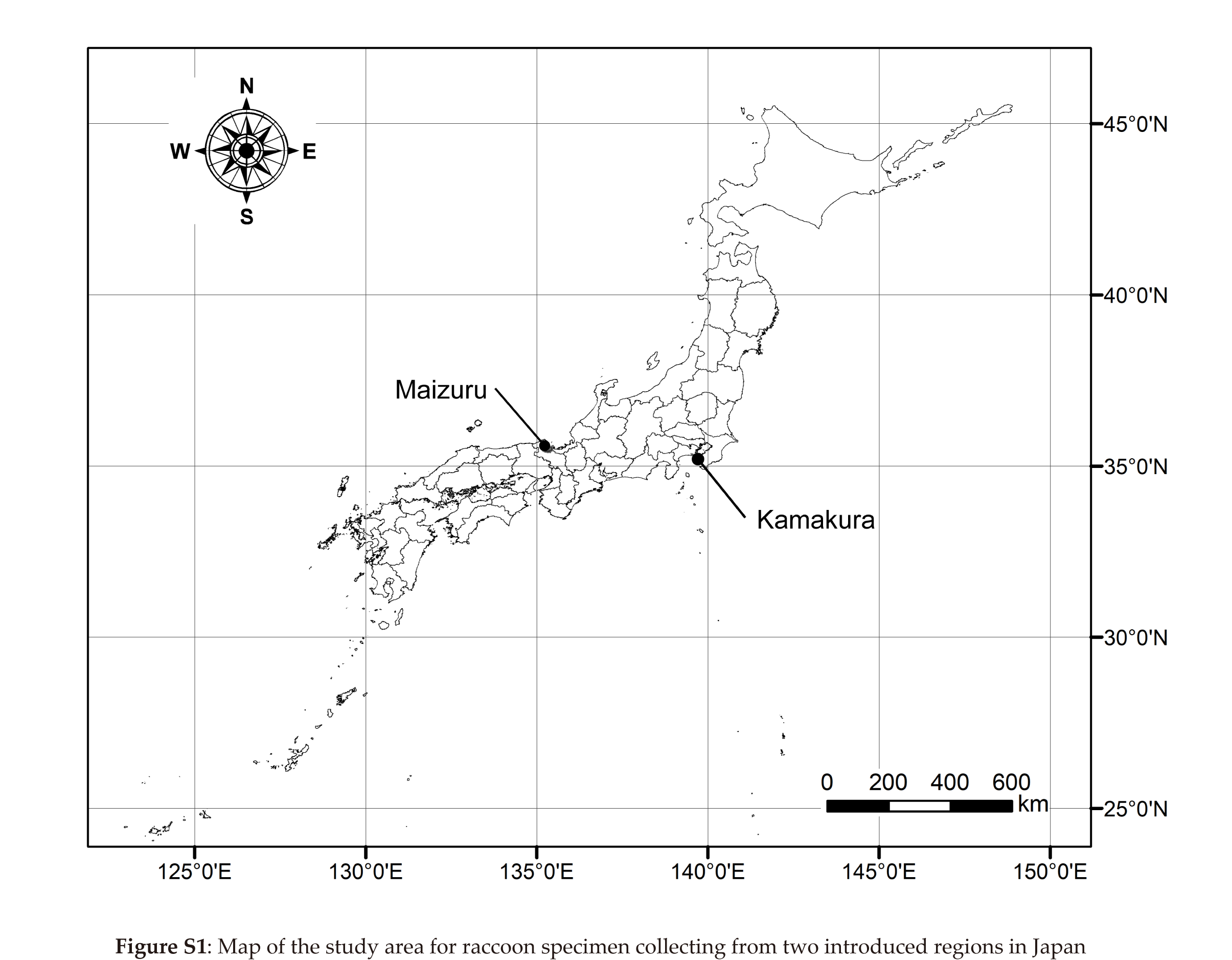

Supplement: Supplementary file 1 [file animals-13-00055-s001.zip › Figure_S1.tif]

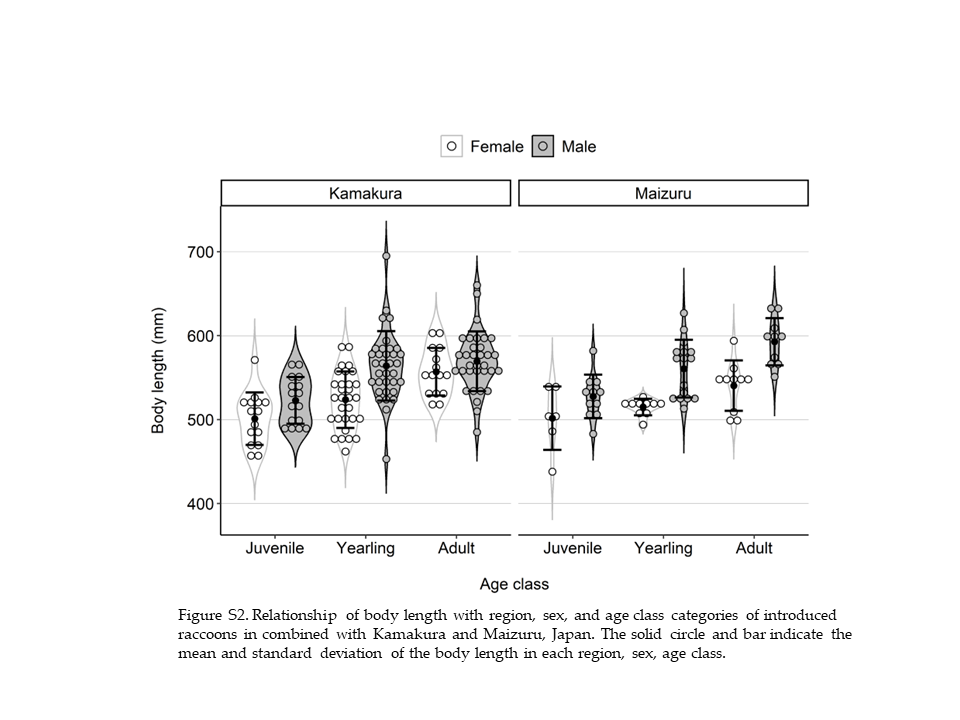

Supplement: Supplementary file 1 [file animals-13-00055-s001.zip › Figure_S2.tif]
